# Efficacy of Dynamic Magnetic Resonance Imaging in the Diagnosis of Degenerative Cervical Myelopathy: Systematic Review Protocol*

**DOI:** 10.1055/s-0044-1779311

**Published:** 2024-03-21

**Authors:** Vanessa Pereira Gil Luizari, Lorena Pereira dos Reis Oliveira, Mariana Demétrio de Sousa Pontes, Thabata Pasquini Soeira, Carlos Fernando Pereira da Silva Herrero

**Affiliations:** 1 Divisão de Fisioterapia, Departamento de Ciências da Saúde, Faculdade de Medicina de Ribeirão Preto, Universidade de São Paulo, Ribeirão Preto, SP, Brasil; 2 Divisão de Fisioterapia, Departamento de Ciências da Saúde, Faculdade de Medicina de Ribeirão Preto, Universidade de São Paulo, Ribeirão Preto, SP, Brasil; 3 Departamento de Ortopedia e Anestesiologia, Doutoranda na Faculdade de Medicina de Ribeirão Preto, Universidade de São Paulo, Ribeirão Preto, SP, Brasil; 4 Divisão de Fisioterapia, Departamento de Ciências da Saúde, Faculdade de Medicina de Ribeirão Preto, Universidade de São Paulo, Ribeirão Preto, SP, Brasil; 5 Departamento de Ortopedia e Anestesiologia, Faculdade de Medicina de Ribeirão Preto, Universidade de São Paulo, Ribeirão Preto, SP, Brasil

**Keywords:** spine, magnetic resonance imaging, cervical spinal cord, compressive myelopathy

## Abstract

Cervical degenerative myelopathy (CDM) is a cervical spine condition resulting in clinical manifestations of spinal cord compression related to the chronic, non-traumatic, and progressive narrowing of the cervical spinal canal. Conventional magnetic resonance imaging (MRI) is the gold standard test to diagnose and assess the severity of CDM. However, the patient is in a neutral and static position during the MRI scan, which may devalue the dynamic factors of CDM, underestimating the risk of spinal cord injury related to cervical spine flexion and extension movements. Dynamic MRI is a promising technique to change this scenario. Therefore, the present review aims to answer the following question: “Is dynamic MRI of the cervical spine more accurate in diagnosing CDM than conventional MRI?”. We will search for studies in the MEDLINE (via PubMed), Embase, Scopus, Web of Science, LILACS, and SciELO databases. The search strategy will contain a combination of terms related to *cervical myelopathy* and *magnetic resonance imaging* . Two independent reviewers will select studies, extract data, and assess the risk of bias. The synthesis of results will be descriptive, considering the main findings of the studies about the outcomes of interest.

## Introduction

The term *degenerative cervical myelopathy* (CDM) represents a series of signs, symptoms, and pathophysiological changes that lead to spinal cord compression in the cervical region. 1 It is most common cause of spinal dysfunction. 2

The clinical manifestations are diverse and result from chronic, non-traumatic, and progressive spinal canal narrowing. 2 Although the role of mechanical compression in CDM is widely known, dynamic factors are also significant. 3 Patients may present with paresthesia in the extremities, decreased dexterity of movements, radicular pain in the upper limb, spasticity, hyperreflexia, ataxia, sphincter dysfunctions, and paresis. 4 Associated conditions, including cervical radiculopathies and arterial disorders, can complicate the diagnosis; this highlights the critical role of the physical examination in cases of clinical suspicion. 5

Magnetic resonance imaging (MRI) is the gold standard test to diagnose and assess the severity of CDM. A complete spinal MRI scan is indicated to prevent other staged lesions from going unnoticed. 5 Recent studies 6 have suggested that, as the patient is in a neutral and static position during the test, conventional MRI may not be able to assess the dynamic factors triggered by the flexion and extension of the cervical spine, which may account for the symptoms. According to the position of the cervical spine, there are descriptions of morphological and pathological variations that only dynamic MRI may identify. 7

### Rationale

The clinical manifestations of CDM are often inconsistent with the findings of conventional MRI scans performed with the patient in a supine position with the neck in a neutral position. 8 This limitation can delay diagnosis and favor the worsening of the disease. 8 Therefore, dynamic MRI has proven to be an essential tool to identify symptoms arising only during cervical spine movements, to define the therapeutic plan, and to increase diagnostic accuracy. 9 10

Hence, the present systematic review aims to synthesize the available evidence on the usefulness of dynamic MRI in diagnosing CDM compared with conventional MRI.

## Materials and Methods

### Research Question

“Is dynamic MRI of the cervical spine more accurate in diagnosing CDM than conventional MRI?”

We defined the systematic review question according to the Population, Intervention, Comparison, and Outcome (PICO) strategy. 11 The included population which will consist of subjects older than 18 years of age, of both genders, with suspected CDM. The intervention will be the performance of a cervical spine dynamic MRI to confirm the diagnostic hypothesis. The comparison will be made with the gold standard test for CDM diagnosis, that is, conventional MRI. And the evaluated outcome will be the potential use of dynamic MRI as the gold standard test to diagnose CDM instead of conventional MRI.

### Eligibility Criteria

The articles selected for the systematic review will be assessed according to the eligibility criteria based on the research question: subjects of both genders, older than 18 years of age, with a suspected diagnosis of DCM, and submitted to a dynamic MRI scan of the cervical spine.

### Information Sources

We will search for studies in the MEDLINE (via PubMed), Embase, Scopus, Web of Science, LILACS, and SciELO databases. To reduce the publication bias, searches will also include the gray literature on Google Scholar, ClinicalTrials.gov, and the OpenGrey platform; in addition, we will analyze the references of the retrieved studies.

### Query Strategy

The search strategy will be based on CDM and dynamic MRI-related terms using the Boolean operators [AND] and [OR]. The search terms will include *degenerative cervical myelopathy* , *cervical myelopathy* , *magnetic resonance imaging* , *dynamic magnetic resonance imaging* , and *MRI* . There will be no restrictions regarding language or year of publication.

Two independent researchers will perform the study searches and record the results regarding the number of articles available in each database in a Microsoft Excel (Microsoft Corp., Redmond, WA, United States), version 15.29, spreadsheet.

### Study Selection and Data Extraction and Registration

Two independent researchers will select the studies using the Mendeley (Elsevier, Amsterdam, The Netherlands) software. In the first selection stage, the evaluators will identify the studies by reading titles and abstracts. The second stage will correspond to the reading of the full text of the articles selected in the first stage. The final selection for the systematic review will include studies meeting the previously-defined eligibility criteria. A third researcher will solve potential disagreements by consensus.

After defining the studies that will form the base of the systematic review, the two evaluators will extract data on general information regarding the publication (year, journal, country), participants (age, gender), designs, and outcomes, also independently, using the Rayyan systematic review manager (Rayyan Systems Inc., Cambridge, MA, United States).

### Analysis of the Methodological Quality of the Studies

The Revised Cochrane Risk of Bias Tool for Randomized Trials (RoB 2) 12 and the Risk of Bias in Non-randomized Studies - of Interventions (ROBINS-I) 13 will be used to assess the risk of bias in randomized clinical trials and observational studies respectively.

### Evidence Quality Assessment

After evaluating the risk of bias, the Grading of Recommendations Assessment, Development, and Evaluation (GRADE) system will be used to determine the quality of the evidence for each outcome. 14

### Data Synthesis

We will descriptively synthesize the information available in the literature and write a systematic review following the Preferred Reporting Items for Systematic Reviews and Meta-Analyses (PRISMA) statement. 15

#### Registration

The systematic review protocol was registered in the International Prospective Register of Systematic Reviews (PROSPERO) 16 database at the University of York (CRD42020221798).

#### Amendments

The present protocol does not represent an amendment to a previously completed or published protocol. If required, records of the protocol amendments will be made in the PROSPERO platform. 16
