# Supplementary material for: Efficacy of Dynamic Magnetic Resonance Imaging in the Diagnosis of Degenerative Cervical Myelopathy: Systematic Review Protocol*
Source: Rev Bras Ortop (Sao Paulo). 2024 Mar 21;59(1):e17–20. doi: 10.1055/s-0044-1779311 (PMC10957276; doi:10.1055/s-0044-1779311)
Supplement: Related texts

# Research article: Eficácia da ressonância magnética dinâmica no diagnóstico da mielopatia cervical degenerativa: Protocolo de revisão sistemática*

**Keywords:** coluna vertebral, imagem por ressonância magnética, medula cervical, mielopatia compressiva

## Abstract

A mielopatia cervical degenerativa (MCD) é uma doença da coluna cervical com manifestações clínicas de compressão da medula espinal relacionadas ao estreitamento crônico, não traumático e progressivo do canal vertebral cervical. A ressonância magnética (RM) convencional é o exame padrão-ouro para o diagnóstico e a avaliação da gravidade da MCD. Contudo, o paciente encontra-se em posição neutra e estática durante a realização deste exame, o que pode desvalorizar os fatores dinâmicos da MCD, subestimando o risco de lesão medular relacionados aos movimentos de flexão e extensão da coluna cervical. A RM dinâmica é uma técnica promissora para modificar esse panorama. Portanto, a presente revisão tem o objetivo de responder a seguinte pergunta: “A RM dinâmica da coluna cervical é mais precisa no diagnóstico de MCD em comparação à RM convencional?” As buscas por estudos serão realizadas nas bases de dados MEDLINE (via PubMed), Embase, Scopus, Web of Science, LILACS e SciELO. A estratégia de busca conterá combinação de termos relacionados à *mielopatia cervical* e à *ressonância magnética* . Dois avaliadores independentes irão realizar a seleção dos estudos, a extração dos dados e a avaliação dos riscos de viés. A síntese dos resultados será realizada de maneira descritiva, considerando os principais achados dos estudos relacionados aos desfechos de interesse.

## Introdução

O termo *mielopatia cervical degenerativa* (MCD) foi introduzido para representar uma série de sinais, sintomas e alterações fisiopatológicas que levam à compressão da medula espinal na região cervical. 1 Esta afecção é a causa mais comum de disfunção da medula. 2

As manifestações clínicas são diversas, e estão relacionadas ao estreitamento crônico, não traumático e progressivo do canal vertebral. 2 Apesar de o papel da compressão mecânica na gênese da MCD já ser amplamente conhecido, fatores dinâmicos também participam desse processo. 3 Os pacientes podem apresentar parestesia nas extremidades, diminuição da destreza dos movimentos, dor radicular no membro superior, espasticidade, hiperreflexia, ataxia, disfunções esfincterianas e paresia. 4 O diagnóstico pode ser dificultado pela presença de condições associadas, como radiculopatias cervicais ou claudicação arterial, sendo indispensável a realização do exame físico minucioso diante da suspeita clínica. 5

A ressonância magnética (RM) é considerada o exame padrão-ouro para o diagnóstico e a avaliação da gravidade da MCD, sendo indicada RM da coluna vertebral completa para evitar que outras lesões estagiadas passem despercebidas. 5 Estudos recentes 6 sugerem que, como o paciente se encontra em posição neutra e estática durante a realização do exame, a RM convencional pode não ser capaz de avaliar os fatores dinâmicos desencadeados pela flexoextensão da coluna cervical, os quais podem ser responsáveis pelos sintomas. Dependendo da posição da coluna cervical, foram descritas variações morfológicas e patológicas que só podem ser identificadas pela RM dinâmica. 7

## Justificativa

A MCD cursa com manifestações clínicas que frequentemente não se relacionam aos achados da RM convencional, a qual é realizada com o paciente em decúbito dorsal com o pescoço em posição neutra. 8 Essa limitação pode retardar diagnósticos e favorecer o agravamento da doença. 8 Por esse motivo, a RM dinâmica tem se mostrado uma ferramenta essencial para auxiliar na identificação de sintomas que surgem apenas com os movimentos da coluna cervical, na definição do plano terapêutico, além de aumentar a precisão dos diagnósticos. 9 10

Diante disso, o objetivo desta revisão sistemática é sintetizar as evidências disponíveis sobre a utilidade da RM dinâmica no diagnóstico da MCD comparada à RM convencional.

## Materiais e Métodos

## Pergunta da Pesquisa

“A RM dinâmica da coluna cervical é mais precisa no diagnóstico de MCD em comparação à RM convencional?”

A pergunta da revisão sistemática foi definida de acordo com a estratégia População, Intervenção, Comparação e Desfecho ( *Outcome* , em inglês) (PICO). 11 A população incluída corresponderá a indivíduos acima de 18 anos, de ambos os sexos, com suspeita de MCD. A intervenção será a realização da RM dinâmica da coluna cervical em busca da confirmação da hipótese diagnóstica. A comparação será feita com o padrão-ouro para o diagnóstico da MCD, ou seja, a RM convencional. E o desfecho avaliado será a possibilidade de se utilizar a RM dinâmica como padrão-ouro no diagnóstico da MCD em vez da RM convencional.

## Critérios de Elegibilidade

Os artigos selecionados para a revisão sistemática serão avaliados de acordo com os critérios de elegibilidade definidos com base na pergunta da pesquisa: indivíduos de ambos os sexos, maiores de 18 anos, com suspeita diagnóstica de MCD e submetidos à RM dinâmica da coluna cervical.

## Fontes de Informação

As buscas por estudos serão realizadas nas bases de dados MEDLINE (via PubMed), Embase, Scopus, Web of Science, LILACS e SciELO.

Para reduzir vieses de publicação, também serão realizadas buscas para incluir a literatura cinzenta no Google Scholar, no ClinicalTrials.gov e na plataforma OpenGrey, além da leitura das listas de referências dos artigos.

## Estratégia de Busca

A estratégia de busca será feita com termos relacionados à MCD e à RM dinâmica, com a utilização dos operadores *booleanos* [AND] e [OR]. Os termos de busca serão: *degenerative cervical myelopathy* , *cervical myelopathy* , *magnetic resonance* , *dynamic magnetic resonance* , e *MRI* . Não haverá restrições de idioma ou de ano de publicação.

As buscas serão feitas por dois pesquisadores independentes e os resultados quanto ao número de artigos disponíveis em cada base de dados serão registrados em uma planilha do programa Microsoft Excel (Microsoft Corp., Redmond, WA, Estados Unidos), versão 15.29.

## Seleção dos Estudos, Extração e Registro dos Dados

A seleção dos estudos será feita pelos dois pesquisadores independentes utilizando o programa Mendeley (Elsevier, Amsterdã, Holanda). Na primeira etapa de seleção, os avaliadores identificarão os estudos pela leitura dos títulos e dos resumos. A segunda etapa corresponderá à leitura completa dos artigos selecionados na primeira. A seleção final para a revisão sistemática incluirá estudos que se adequarem aos critérios de elegibilidade previamente definidos. As possíveis discordâncias serão solucionadas por consenso com um terceiro pesquisador.

Uma vez definidos os estudos que farão parte da revisão sistemática, os dois avaliadores farão a extração dos dados referentes às informações gerais da publicação (ano, periódico, país), aos participantes (idade, gênero), aos desenhos e aos resultados dos estudos, também de maneira independente, por meio do gerenciador de revisão sistemática Rayyan (Rayyan Systems Inc., Cambridge, MA, Estados Unidos).

## Análise da Qualidade Metodológica dos Estudos

Os riscos de viés serão avaliados utilizando as ferramentas Revised Cochrane Risk of Bias Tool for Randomized Trials (RoB 2) 12 e Risk of Bias in Non-randomized Studies *-* of Interventions (ROBINS-I), 13 para ensaios clínicos randomizados e para estudos observacionais, respectivamente.

## Avaliação da Qualidade das Evidências

Após a avaliação dos riscos de viés, a avaliação da qualidade das evidências para cada um dos desfechos será feita utilizando-se o sistema Grading of Recommendations Assessment, Development, and Evaluation (GRADE). 14

## Síntese dos Dados

A síntese da informação disponível na literatura será redigida em forma descritiva, e a revisão sistemática será escrita de acordo com as normas da declaração Preferred Reporting Items for Systematic Reviews and Meta-Analyses (PRISMA). 15

## Registro

O registro do protocolo da revisão sistemática foi feito no banco de dados International Prospective Register of Systematic Reviews (PROSPERO), 16 da Universidade de York (CRD42020221798).

## Emendas

Este protocolo não representa uma alteração de um protocolo previamente concluído ou publicado. Se necessário, a documentação de emendas ao protocolo será feita na plataforma PROSPERO. 16
